# A Novel Hybrid Approach for UT1-UTC Ultra-Short-Term Prediction Utilizing LOD Series and Sum Series of LOD and First-Order-Difference UT1-UTC

**DOI:** 10.3390/s25041087

**Published:** 2025-02-11

**Authors:** Fei Ye, Minsi Ao, Ningbo Li, Rong Zeng, Xiangqiang Zeng

**Affiliations:** BeiDou High-Precision Satellite Navigation and Location Service Hunan Engineering Research Center, Hunan Institute of Geomatics Sciences and Technology, Shaoshanzhong Road No. 693, Changsha 410007, China

**Keywords:** UT1-UTC ultra-short-term prediction, novel hybrid method, LOD, LS + AR, LS + MAR, sum of LOD and first-order-difference UT1-UTC

## Abstract

Accurate ultra-short-term prediction of UT1-UTC is crucial for real-time applications in high-precision reference frame conversions. Presently, traditional LS + AR and LS + MAR hybrid methods are commonly employed for UT1-UTC prediction. However, inherent unmodeled errors in fitting residuals of these methods often compromise the prediction performance. Thus, mitigating these common unmodeled errors presents an opportunity to enhance UT1-UTC prediction performance. Consequently, we propose a novel hybrid difference method for UT1-UTC ultra-short-term prediction by integrating LOD prediction and the prediction of the sum of the LOD and the first-order-difference UT1-UTC. The evaluation demonstrated promising results: (1) The mean absolute errors (MAEs) of the proposed method range from 21 to 869 µs in 1–10-day UT1-UTC predictions. (2) Comparative analysis against zero-/first-/second-order-difference LS + AR and zero-/first-order-difference LS + MAR hybrid method reveals a substantial reduction in MAEs by an average of 54/64/44 µs, and 47/20 µs, respectively, with the proposed method. (3) Correspondingly, the proposed method achieves average improvement percentages of 17%/18%/15%, and 13%/3% in 1–10-day UT1-UTC predictions.

## 1. Introduction

The Earth rotation parameters (ERPs), encompassing diurnal rotation typically denoted as the difference (UT1-UTC) between UT1 and Coordinated Universal Time (UTC), are pivotal for converting between celestial and terrestrial reference systems, crucial for tasks such as the precise orbit determination of Global Navigation Satellite System (GNSS) satellites [1]. Among rotation parameters, UT1-UTC poses challenges in real-time estimation due to time delays inherent in data processing via space geodetic techniques like very long baseline interferometry (VLBI) [2,3,4]. Consequently, enhancing UT1-UTC ultra-short-term predictions (1–10 days) is imperative for real-time applications pertaining to reference frames, such as rapid and ultra-rapid orbit determination of GNSS satellites [5].

Considerable research has been devoted to ERP prediction methods and their performance evaluation. The least squares (LS) method [6] is a prevalent approach for fitting ERP data, supplemented by Kalman-filter-based methods [7,8] and hybrid methodologies like LS + AR (autoregressive) [9,10,11], LS + MAR (multivariate autoregressive) [12,13], LS + AR + Kalman filter [14], and LS + MAR + Kalman filter [15]. Furthermore, neural network [16], fuzzy-wavelet-based prediction [17], copula-based analysis, and singular spectrum analysis (SSA) methods [18] are deployed for ERP prediction. Additionally, hybrid techniques integrating the gray model with neural network or autoregressive integrated moving average (ARIMA) are utilized [19]. Among these, extensive efforts have been made to enhance the LS + AR hybrid method’s prediction performance [20,21,22], including weight design [23], simultaneous parameter calculation for LS and AR models [24], and series differencing [25]. Moreover, the prediction performance of these methods can be further enhanced by leveraging additional effective angular momentum (EAM) [26,27]. While acknowledging their potential, such as EAM and weight considerations, it can be extended them to the prediction method.

Furthermore, global Earth rotation parameter (ERP) predictions have been conducted and compared by the Vienna University of Technology and the International Earth Rotation and Reference Systems Service (IERS) to assess the performance of various ERP prediction methods [9,28]. While the LS + AR hybrid method is among the main methods recommended for ultra-short-term ERP prediction, no single method is universally suitable for all ERP prediction lengths [29], prompting the recommendation for combined algorithms to achieve optimal performance [30,31]. Therefore, proposing an effective novel method holds promise for contributing to the ultimate combined solution for UT1-UTC prediction, underscoring the importance of devising high-performance methods [27].

Moreover, it has been established that the UT1-UTC prediction performance can be enhanced by incorporating length of day (LOD) information into the LS + MAR hybrid method [13,32]. The IERS Rapid Service/Prediction Centre utilizes LOD information in their UT1-UTC predictions (https://www.iers.org/, accessed on 26 November 2024). Given the strong relationship between UT1-UTC and LOD, traditional methods like LS + AR and LS + MAR may exhibit common unmodeled errors originating from the same excitation sources in the fitting residuals of LOD and first-order-difference UT1-UTC. However, these errors can be mitigated or eliminated in their sum series, aligning more closely with stable random characteristics and yielding better performance in autoregressive (AR) models. Additionally, LOD boasts superior observation accuracy compared to UT1-UTC (https://www.iers.org/, accessed on 26 November 2024) and can be directly observed using GNSS techniques, with its time delay estimated by GNSS techniques shorter than that estimated by very long baseline interferometry (VLBI) (http://www.igs.org/, accessed on 26 November 2024; http://www.igmas.org/, accessed on 26 November 2024) [33].

This suggests that considering the prediction of LOD, along with the prediction of the sum of LOD and first-order-difference UT1-UTC, can enhance the performance of UT1-UTC ultra-short-term prediction. Building upon this concept, we propose a novel hybrid difference method for UT1-UTC ultra-short-term prediction and evaluate its performance by applying the LS + AR hybrid method to predict the LOD. This study commences with a description of the proposed method, followed by the implementation and analysis of its prediction performance. Finally, conclusions are drawn based on the findings.

## 2. Novel Hybrid Difference Method for UT1-UTC Ultra-Short-Term Prediction

**Figure 1:**
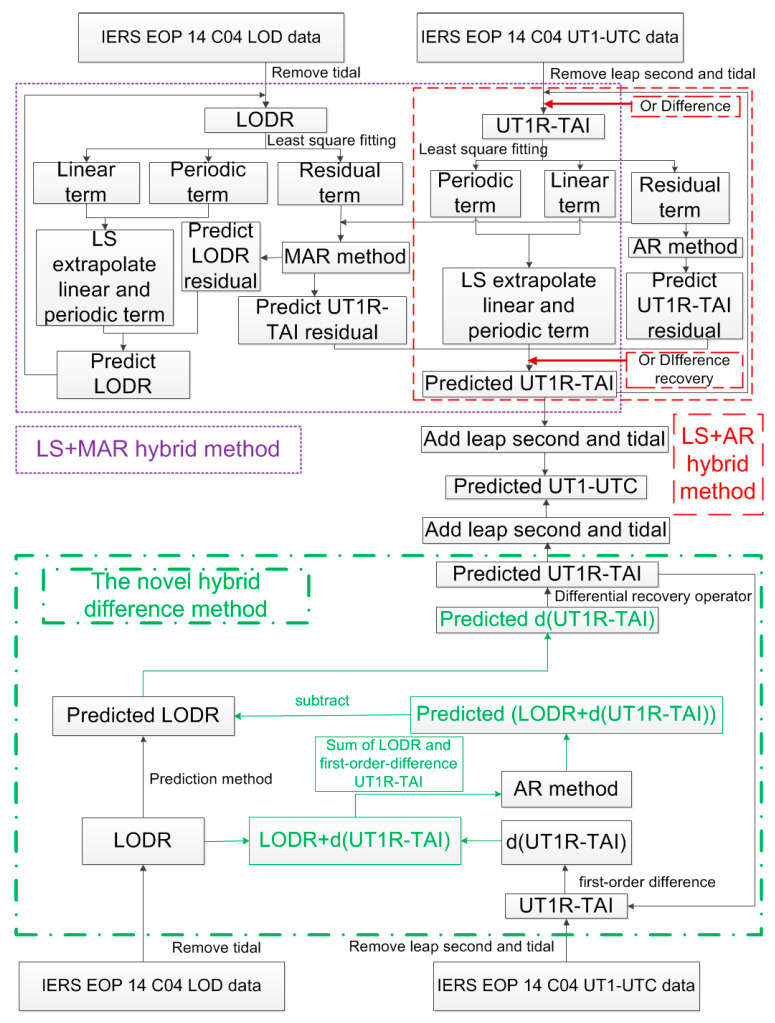
Details of the novel hybrid difference method and the traditional LS + AR hybrid and LS + MAR hybrid methods. Red indicates the traditional LS + AR hybrid method; purple indicates the traditional LS + MAR hybrid method; and green indicates the novel hybrid difference method.

The LS + AR and LS + MAR methods have been demonstrated in Xu et al. [14] and Tan et al. [15]. The UT1R-TAI (UT1R: UT1 minus tidal effects, TAI: International Atomic Time) and LODR (LOD minus Earth’s zonal harmonic tidal) series, which are applied to the hybrid method, are obtained after preprocessing [34]. Here, the prediction value of the first-order-difference UT1R-TAI is expressed as

(1) $$\Delta {\overset{\wedge }{f}}_{t+1}^{UT1R-TAI}={\overset{\wedge }{Z}}_{AR,t+1}^{\Delta \left(UT1R-TAI\right)+LODR}-{\overset{\wedge }{f}}_{t+1}^{LODR},$$

where $\Delta {\overset{\wedge }{f}}_{t+1}^{UT1R-TAI}$ refers to the prediction value of the first-order-difference UT1R-TAI at time *t* + 1; ${\overset{\wedge }{Z}}_{AR,t+1}^{\Delta \left(UT1R-TAI\right)+LODR}$ is the prediction value of the autoregressive (AR) model for the sum of the LODR and the first-order-difference UT1R-TAI at time *t* + 1; and ${\overset{\wedge }{f}}_{t+1}^{LODR}$ stands for the prediction value of LODR at time *t* + 1. Then, the predicted UT1R-TAI ${\overset{\wedge }{f}}_{t+1}^{UT1R-TAI}$ at time *t* + 1 is recovered by

(2) $${\overset{\wedge }{f}}_{t+1}^{UT1R-TAI}=\Delta {\overset{\wedge }{f}}_{t+1}^{UT1R-TAI}+f_{t}^{UT1R-TAI},$$

where $f_{t}^{UT1R-TAI}$ is the observed value of UT1R-TAI at time t. Finally, the predicted UT1-UTC at time *t* + 1 can be obtained by adding the tidal effects and leap second for ${\overset{\wedge }{f}}_{t+1}^{UT1R-TAI}$. The details of the novel hybrid difference method and the traditional LS + AR and LS + MAR hybrid methods for UT1-UTC ultra-short-term prediction are depicted in Figure 1.

## 3. Results

To analyze and evaluate the UT1-UTC ultra-short-term prediction (1–10 days) performance of the novel hybrid difference method, six solutions were designed, and these six cases are described as follows:

Case LS + AR: LS + AR hybrid method based on the UT1-UTC data series, namely the traditional LS + AR hybrid method [9];
Case First-diff LS + AR: LS + AR hybrid method based on the first-order-difference UT1-UTC data series, namely the traditional first-order-difference LS + AR hybrid method [25];
Case Second-diff LS + AR: LS + AR hybrid method based on the second-order-difference UT1-UTC data series, namely the traditional second-order-difference LS + AR hybrid method [35];
Case LS + MAR: LS + MAR hybrid method based on the UT1-UTC and LOD data series, namely the traditional LS + MAR hybrid method [12,32];
Case First-diff LS + MAR: LS + MAR hybrid method based on the first-order-difference UT1-UTC and LOD data series, namely the traditional first-order-difference LS + MAR hybrid method [13];
Case new method: The novel hybrid difference method based on the UT1-UTC and LOD data series.

Data description: The UT1-UTC/LOD data series of the IERS EOP 14 C04 product spans from 1 January 2010, (MJD: 55197) to 16 May 2021 (MJD: 59350). Specifically, the data series from 19 October 2020, (MJD: 59141) to 16 May 2021, (MJD: 59350) are utilized as the reference values. The total length of all predictions is 210 days, covering the period from 19 October 2020, (MJD: 59141) to 16 May 2021 (MJD: 59350). Each prediction is conducted iteratively on a daily basis, spanning 1–10 days per prediction, repeated for a total of 210 times. In these experiments, we define

(3) $${\overset{\wedge }{f}}_{t+1}^{LODR}={\overset{\wedge }{f}}_{t+1}^{\mathrm{LS}}+{\overset{\wedge }{Z}}_{t+1}^{AR},$$

where ${\overset{\wedge }{f}}_{t+1}^{\mathrm{LS}}$ is the LS extrapolation value of the LS model for LODR, and ${\overset{\wedge }{Z}}_{t+1}^{AR}$ is the prediction value of the AR model for the LS fitting residuals of LODR.

**Figure 2:**
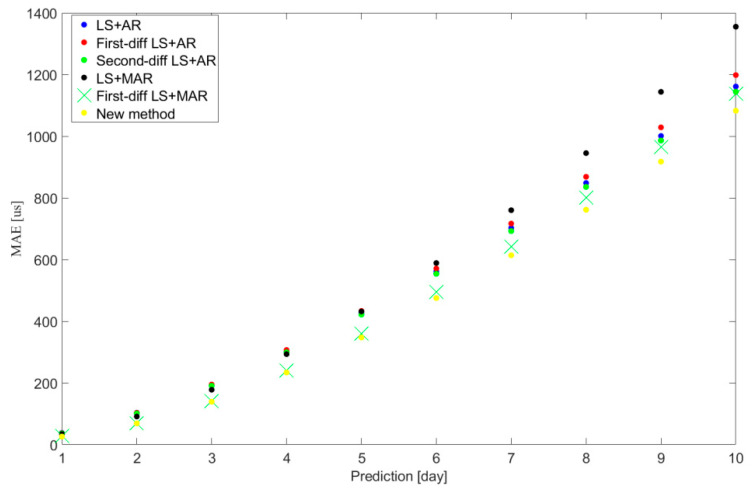
Mean absolute errors (MAEs) of UT1-UTC ultra-short-term prediction for six different cases. Blue represents case LS + AR, red represents case First-diff LS + AR, green represents case Second-diff LS + AR, black represents case LS + MAR, green X represents case First-diff LS + MAR, and yellow represents case new method.

The UT1-UTC ultra-short-term predicted results of cases LS + AR, First-diff LS + AR, Second-diff LS + AR, LS + MAR, First-diff LS + MAR, and new method are illustrated in Figure 2. Furthermore, Table 1 presents the mean absolute errors (MAEs) [9] and relevant statistics for these six cases.

Figure 2 illustrates that the proposed method outperforms the traditional LS + AR hybrid methods, whether based on zero-order-difference, first-order-difference, or second-order-difference UT1-UTC data series. Additionally, it shows superior performance compared to the traditional LS + MAR hybrid method using LOD and zero-order-difference or first-order-difference UT1-UTC data series.

Table 1 provides detailed insights, indicating that the mean absolute errors (MAEs) of the UT1-UTC ultra-short-term predictions for the proposed method range from 21 to 869 µs over 1–10 days. Comparatively, the proposed method reduces MAEs by 7–76 µs, translating to improvement percentages of 8–28% compared to the traditional LS + AR hybrid method. Similarly, compared to the traditional first-order-difference LS + AR hybrid method, the MAEs are reduced by 6–109 µs, with improvement percentages ranging from 11% to 28%. Moreover, compared to the traditional second-order-difference LS + AR hybrid method, the MAEs decrease by 5–55 µs, resulting in improvement percentages of 6–26%. Furthermore, compared to the traditional LS + MAR hybrid method, the MAEs are reduced by 5–107 µs, with improvement percentages ranging from 10% to 20%. Finally, in comparison to the first-order-difference LS + MAR hybrid method [13], the MAEs decrease by 1–65 µs, with improvement percentages ranging from 1% to 7% over 1–10 days.

## 4. Discussion

**Figure 3:**
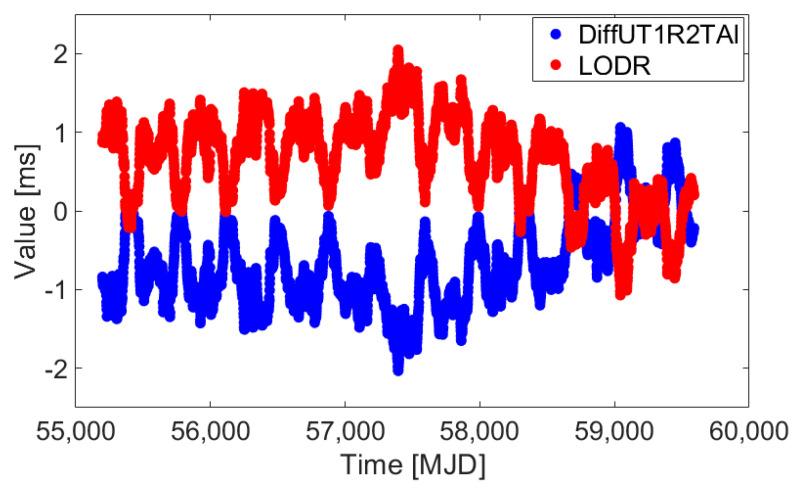
LODR and first-order-difference UT1R-TAI series from 1 January 2010, to 6 May 2021. Red represents the LODR series, while blue represents the first-order-difference UT1R-TAI series. Their correlation coefficient is −0.9989.

**Figure 4:**
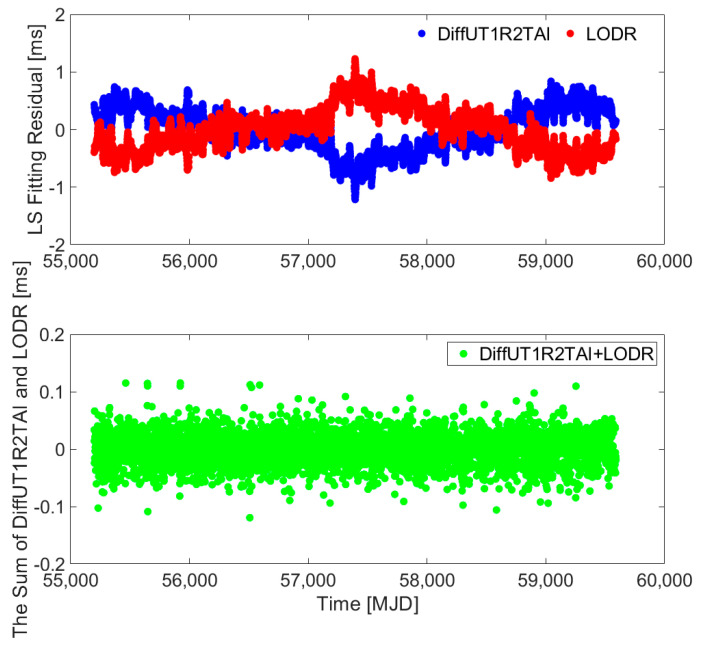
LS fitting residuals of LODR and first-order-difference UT1R-TAI from 1 January 2010, to 6 May 2021, (**top**) and the sum series of LODR and first-order-difference UT1R-TAI (**bottom**). Blue represents the LS fitting residual of the first-order-difference UT1R-TAI, red represents the LS fitting residual of LODR, and green represents the sum series of LODR and first-order-difference UT1R-TAI.

To further discuss and verify the reliability of the novel hybrid difference method, the LODR, first-order-difference UT1R-TAI, and the LS fitting residuals of the LODR and the first-order-difference UT1R-TAI are displayed. The UT1-UTC/LOD data series of the EOP (Earth Orientation Parameters) 14 C04 product from 1 January 2010, (Modified Julian Date, MJD: 55197) to 6 May 2021, (MJD: 59340) are provided by the IERS (International Earth Rotation and Reference Systems Service). After preprocessing [36], the LODR and first-order-difference UT1R-TAI series are depicted in Figure 3, while their sum series are illustrated in the top panel of Figure 4. The LS fitting residuals for LODR and first-order-difference UT1R-TAI are described in the bottom panel of Figure 4.

Figure 3 shows that the LODR and first-order-difference UT1R-TAI series have regular symmetry around zero. This can be explained by the fact that they are affected by same excitation source and force, and it is consistent with their definition. In addition, their correlation coefficient is −0.9989, which also indicates that they are strongly correlated but not precisely equivalent in terms of these data.

In addition, Figure 3 illustrates that some common unmodeled errors for fitting residuals still exist, and this may reduce the performance of UT1-UTC ultra-short-term prediction. In addition, as Figure 4 shows, the sum series of the LODR and first-order-difference UT1R-TAI has more obvious zero-mean characteristics of stable random distribution compared to the LS fitting residuals of LODR and first-order-difference UT1R-TAI, which also indicates agreement with the above sections.

## 5. Conclusions

We introduced a novel hybrid difference method aimed at enhancing the performance of UT1-UTC ultra-short-term prediction by incorporating LOD information. By leveraging the relationship between the LOD and first-order-difference UT1-UTC, we mitigate the unmodeled errors common in traditional methods. Our proposed method was evaluated against traditional hybrid methods using IERS EOP 14 C04 UT1-UTC and LOD data spanning from 1 January 2010, to 6 May 2021. The findings regarding UT1-UTC ultra-short-term prediction are summarized as follows:

Compared to traditional methods such as LS + AR hybrid, first-order-difference LS + AR hybrid, second-order-difference LS + AR hybrid, LS + MAR hybrid, and first-order-difference LS + MAR hybrid method, the proposed method exhibits reductions in the mean absolute errors (MAEs) ranging from 7 to 76 µs, 6 to 109 µs, 5 to 56 µs, 5 to 107 µs, and 1 to 65 µs, respectively, across predictions spanning 1–10 days. The improvement percentages range from 8% to 28%, 11% to 28%, 6% to 26%, 10% to 20%, and 1% to 7%, respectively. This underscores the efficacy of our proposed method, which considers both the LOD prediction and the prediction of the sum of the LOD and the first-order-difference UT1-UTC in enhancing the performance of ultra-short-term UT1-UTC prediction.

In addition, the proposed method utilizes the prediction of the LOD and the prediction of the sum of the LOD and the first-order-difference UT1-UTC; so, the UT1-UTC prediction performance will be further enhanced if the prediction performance of LOD or the prediction performance of the sum of LOD and the first-order-difference UT1-UTC is improved.

## Figures and Tables

**Table 1:** Mean absolute errors (MAEs) of the UT1-UTC ultra-short-term prediction for cases LS + AR, First-diff LS + AR, Second-diff LS + AR, LS + MAR, First-diff LS + MAR, and new method (units: µs). IMP1, IMP2, IMP3, IMP4, and IMP5 indicate the improvement of the new method over case LS + AR, First-diff LS + AR, Second-diff LS + AR, LS + MAR, and First-diff LS + MAR, respectively.

Prediction Day/Cases	1	2	3	4	5	6	7	8	9	10
LS + AR	28	78	150	242	344	456	576	694	817	942
First-diff LS + AR	27	78	152	245	349	462	583	709	840	978
Second-diff LS + AR	26	76	149	239	339	445	558	673	796	922
LS + MAR	26	70	134	218	320	434	558	692	831	976
First-diff LS + MAR	21	56	112	191	291	403	526	657	794	934
New method	21	56	111	191	286	390	502	619	741	869
IMP1 (%)	25	28	26	21	17	14	13	11	9	8
IMP2 (%)	22	28	27	22	18	16	14	13	12	11
IMP3 (%)	19	26	26	20	16	12	10	8	7	6
IMP4 (%)	19	20	17	12	11	10	10	11	11	11
IMP5 (%)	0	0	1	0	2	3	5	6	7	7

## Data Availability

The UT1–UTC/LOD data products from IERS are available at https://www.iers.org/IERS/EN/DataProducts/EarthOrientationData/eop.html, accessed on 26 November 2024.
